# Cephalometric evaluation of changes in vertical dimension and molar position in adult non-extraction treatment with clear aligners and traditional fixed appliances

**DOI:** 10.1590/2177-6709.26.4.e2119360.oar

**Published:** 2021-09-10

**Authors:** Hailee RASK, Jeryl D. ENGLISH, Clark COLVILLE, Fred Kurtis KASPER, Ronald GALLERANO, Helder Baldi JACOB

**Affiliations:** 1 The University of Texas Health Science Center at Houston School of Dentistry, Department of Orthodontics (Houston/TX, USA).

**Keywords:** Clear aligner, Traditional fixed appliance, Vertical dimension, Molar height

## Abstract

**Introduction:**

Orthodontists have been using clear aligners to treat malocclusions, and one potential effect of treatment with orthodontic aligners is the intrusion and/or resists extrusion of the posterior teeth. This “bite-block effect” is primarily anecdotal due to the frequent occurrence of posterior open bites in patients after clear aligner therapy.

**Objective:**

The purpose of this study was to compare changes promoted by clear aligners and traditional fixed appliances in cephalometric measurements of the vertical dimension and molar position in adult patients with Class I malocclusion treated with non-extraction.

**Methods:**

Pre- and post-treatment lateral cephalometric radiographs of adult patients treated with either clear aligners (n=44) or traditional fixed appliances (n=22) were selected for retrospective analysis. Eight interval measurements and one nominal measurement were evaluated: anterior overbite (OB), mandibular plane angle related to cranial base (SN_MP) and related to Frankfort (FMA), lower molar height (L6H) and upper molar height (U6H), palatal plane to mandibular plane angle (PP_MP), lower facial height (LFH), total facial height (TFH), and posterior open bite (Posterior_OB). A single evaluator traced all cephalographs, and changes in select measures of the vertical dimension were compared within and between groups.

**Results:**

OB decreased (1.15 mm) and L6H increased (0.63 mm) in the traditional fixed appliance group. Mandibular plane angles (related to cranial base and to Frankfort) increased (0.43° and 0.53°, respectively) in the clear aligner group, but just FMA showed significant difference between groups (difference of 0.53°). LFH and TFH increased (ranging from 0.52 mm to 0.80 mm) in both groups, with no differences between treatment modality. Presence of visible posterior open bite significantly increased over the course of treatment. OB, FMA and L6H exhibited an interaction between treatment stage (pre- and post-treatment) and modality (clear aligner therapy and traditional fixed appliances), but no interaction among these three variables was found.

**Conclusions:**

The evidence does not support the theory that clear aligner therapy produces better vertical dimension control than traditional fixed appliances. Traditional fixed appliance therapy slightly extruded the lower molar, and clear aligner therapy produced a slightly mandibular backward rotation.

## RESUMO

**Introdução:**

Ortodontistas têm usado os alinhadores transparentes para tratar más oclusões, e um potencial efeito desse tratamento é a intrusão e/ou resistência à extrusão dos dentes posteriores. Esse efeito de “bloco de mordida” é principalmente empírico, devido à ocorrência frequente de mordidas abertas posteriores em pacientes após a terapia com alinhadores transparentes.

**Objetivo:**

O objetivo do presente estudo foi comparar as mudanças promovidas pelos alinhadores transparentes e aparelho fixo convencional nas medidas cefalométricas de dimensão vertical e posição do molar em pacientes adultos com má oclusão de Classe I tratados sem exodontias.

**Métodos:**

Radiografias cefalométricas laterais pré- e pós-tratamento de pacientes adultos tratados com alinhadores transparentes (n=44) ou com aparelho fixo tradicional (n=22) foram selecionadas para uma análise retrospectiva. Oito medidas de intervalo e uma medida nominal foram avaliadas: trespasse vertical anterior (OB), ângulo do plano mandibular em relação à base do crânio (SN_MP) e em relação ao Plano de Frankfurt (FMA), altura do molar inferior (L6H) e altura do molar superior (U6H), ângulo do plano palatal ao plano mandibular (PP_MP), altura facial inferior (LFH), altura facial total (TFH) e mordida aberta posterior (Posterior_OB). Um único avaliador fez todos os traçados cefalométricos, e as mudanças nas medidas da dimensão vertical foram comparadas intra e intergrupos.

**Resultados:**

OB reduziu (1,15 mm) e L6H aumentou (0,63 mm) no grupo de aparelho fixo tradicional. Os ângulos do plano mandibular (em relação à base do crânio e ao plano de Frankfurt) aumentaram (0,43° e 0,53°, respectivamente). No grupo dos alinhadores invisíveis, apenas o FMA apresentou diferença significativa entre os grupos (diferença de 0,53º). LFH e TFH aumentaram (variando de 0,52 mm a 0,80 mm) em ambos os grupos, sem diferenças entre as modalidades de tratamento. A presença de uma mordida aberta posterior visível aumentou significativamente durante o curso do tratamento. OB, FMA e L6H exibiram interação entre o estágio do tratamento (pré- e pós-tratamento) e a modalidade (terapia com alinhadores invisíveis ou aparelho fixo tradicional), porém não foi encontrada interação entre essas três variáveis.

**Conclusões:**

A evidência não suporta a teoria de que a terapia com alinhadores invisíveis produz melhor controle da dimensão vertical do que o aparelho fixo. O tratamento com aparelhagem fixa extruiu ligeiramente o molar inferior, e o tratamento com alinhadores invisíveis produziu uma ligeira rotação posterior da mandíbula.

## INTRODUCTION

Orthodontists have traditionally focused on anteroposterior dentoskeletal relationships, but many malocclusions are due to abnormal vertical development. Vertical control during orthodontic treatment has been a challenging problem in orthodontics.^1^ ^,^ ^2^ Therefore, it is often an objective to maintain or decrease vertical dimension in orthodontic patients, especially in hyperdivergent partients.^1^ ^,^ ^3^ Successful treatment depends on the orthodontist’s ability to control vertical tooth movements, because the extrusion of the posterior teeth is the main etiology of the unwanted side effects, such as backward mandibular rotation.^1^ ^,^ ^4^

In the past, orthodontists have traditionally addressed the vertical dimension of patients with high-pull headgear, both with and without extractions, but this approach appears to have little or no effect on the anteroposterior position of the mandible.^5^ ^-^ ^7^ Today, some orthodontists may even attempt to reduce the mandibular plane angle, producing forward mandibular rotation using miniplates^8^ and miniscrews.^1^ Orthodontists know that fixed appliance therapy tends to extrude teeth, increasing the mandibular plane angle.^3^

Recently, orthodontists have been using clear aligners to treat malocclusions due to esthetics, convenience, and comfort.^9^ ^,^ ^10^ Additionally, as the materials and techniques advance, more cases can be adequately treated with aligners.^11^ ^-^ ^13^ One potential effect of treatment with orthodontic aligners is the “bite-block effect”. In theory, the thickness of the aligner plastic combined with occlusal forces leads to intrusion and/or resists extrusion of the posterior teeth over the course of treatment. Evidence for this “bite-block effect” is primarily anecdotal and substantiated by the frequent occurrence of posterior open bites in patients after clear aligner therapy.^11^ ^,^ ^14^ ^,^ ^15^ Some practitioners even recommend clear aligner therapy for patients who present with anterior open bite tendency, due to this alleged benefit.^16^ ^,^ ^17^ As demand for clear aligner therapy increases, it is imperative for the orthodontist to understand how they act on the oral system.

Both traditional fixed appliances and clear aligners work by applying forces to teeth. Despite utilizing the same principles, there are many differences between the treatment modalities. A key difference is the ability to remove orthodontic aligners, which makes patient compliance imperative. Another significant difference is the appliance design. Aligners are polymer trays that fit snugly around the teeth, allowing force application from various directions, as opposed to traditional braces, which act primarily through the bracket on the buccal surface.^18^ This difference leads to a number of advantages (e.g. patient comfort) ^10^ and disadvantages (e.g. limitations in amount of movement per aligner) associated with clear aligner therapy.^11^

It is important to investigate these claims and understand exactly how clear aligner therapy affects the vertical dimension in adult patients (the primary population requesting aligner therapy). If clear aligner therapy does limit changes in the vertical dimension or provides true intrusion, it could become a valuable treatment tool. However, if intrusion is not occurring, then the familiar post-treatment posterior open bite is due to other occurrences such as anterior interferences. It is the responsibility of orthodontists to understand the effects of their appliances, to provide the highest quality treatment results.

The aim of this longitudinal retrospective study is to compare changes in cephalometric measurements that represent the vertical dimension and molar position, before and after treatment in adult patients with Class I malocclusion treated with non-extraction, single-phase comprehensive treatment using clear aligners (Align Technology, Santa Clara, CA, USA) and traditional fixed edgewise appliances (Forestadent, Pforzheim, Germany). The null hypothesis was that there are no differences between treated groups.

## MATERIAL AND METHODS

### SAMPLE SELECTION

This observational retrospective, longitudinal, study used pre- and post-treatment lateral cephalometric radiographs from a sample of patients treated in a private practice, in Seguin, Texas. All patients were treated by the same orthodontist, and they were offered two modalities of treatment, clear aligner and traditional fixed appliance, according to the orthodontist office’s policy. Patients were chosen based on the following inclusion criteria: Class I malocclusion, non-growing (18 years of age or older and cervical vertebral maturation stage V at beginning of treatment), mild to moderate crowding (6 mm or less per arch), no planned molar intrusion or extrusion (vertical movement of the molars in the final accepted ClinCheck was set and maintained at 0.0 mm for the entire series of aligners). Deep bite and crowding were treated using relative intrusion (proclination of the incisors) and interproximal reduction. Patients were excluded if they presented with the following criteria: congenital syndromes, crowding requiring extractions, and missing teeth other than third molars. In addition, radiographs that were not of diagnostic quality (i.e. improper patient positioning or lacking a ruler for calibration) were not utilized. Based on these parameters, 44 patients treated with clear aligners (27 females and 17 males, averaging 41.26 ± 14.59 years of age) and 22 patients treated with traditional fixed appliances (16 females and 6 males, averaging 32.01 ± 11.81 years of age) were identified. All patients had completed treatment within 2 years of May 2019.

### CEPHALOMETRIC ANALYSIS

Standard lateral cephalometric radiographs of the selected patients were recorded at two stages: pre-orthodontic treatment (T_1_) and immediately after orthodontic treatment (T_2_). The traditional fixed appliances (brackets) patients were treated with bi-dimensional 0.018 x 0.022-in Edgewise brackets. The clear aligner patients were treated with Invisalign. Participants were monitored every 6-8 weeks. 

**Figure 1:**
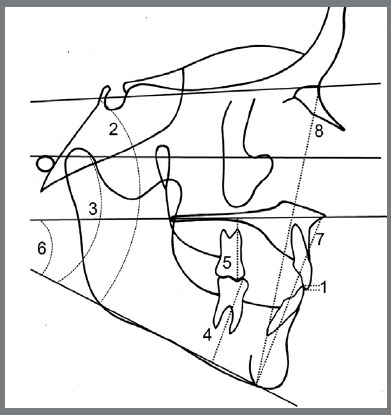
Cephalometric measurements evaluated: 1) OB; 2) SN_MP; 3) FMA; 4) L6H; 5) U6H; 6) PP_MPA; 7) LFH; 8) TFH.

**Figure 2:**
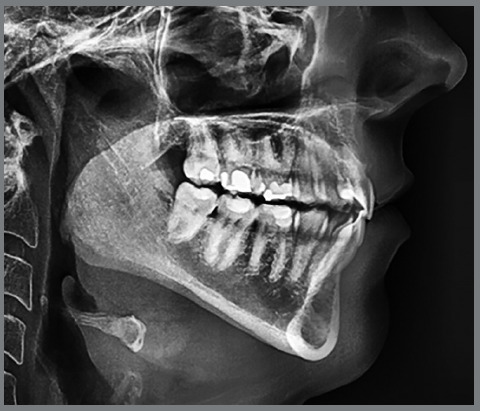
Lateral cephalometric radiograph showing a patient presenting a posterior open bite (lack of posterior occlusal contact).

The pre- and post-treatment lateral cephalograms were traced according to the American Board of Orthodontists (ABO guidelines^19^ using Quick Ceph Software (Quick Ceph Systems, Inc., San Diego, CA) by one of the authors, who was blinded to the treatment stage and modality. A ruler positioned in each radiograph was used to adjust image size. The evaluator also superimposed each subject’s records to ensure accurate tracing and magnification adjustments. Eight vertical measures (Table 1) were recorded for each lateral cephalogram (Fig 1): overbite (OB), sella-nasion to mandibular plane angle (SN_MP), Frankfort to mandibular plane angle (FMA), lower molar height (L6H), upper molar height (U6H), palatal plane to mandibular plane (PP_MP), lower facial height (LFH), and total anterior facial height (TFH). The evaluator also recorded whether posterior open bites [posterior_OB (visible space between the posterior teeth)] were seen on each lateral cephalogram (Fig 2). The posterior_OB was described as yes or no (lack of occlusal contact between maxillary and mandibular molars or contact between maxillary and mandibular molars, respectively).

**Table 1:** Definition of measurements.

Overbite (OB)	Distance (mm) measured from incisal edge of the most anterior upper central incisor (U1) to incisal edge of the most anterior lower central incisor (L1).
Sella-Nasion to Mandibular Plane Angle (SN_MP)	Angle (degrees) measured between sella-nasion (SN) plane and mandibular plane (Go-Me).
Frankfort to Mandibular Plane Angle (FMA)	Angle (degrees) measured between Frankfort Horizontal (Po-Or) and mandibular plane (Go-Me).
Lower Molar Height (L6H)	Distance (mm) measured perpendicular of line from mandibular plane (Go-Me) to mesial-buccal (MB) cusp of lower first molar (L6).
Upper Molar Height (U6H)	Distance (mm) measured perpendicular of line from palatal plane (ANS-PNS) to mesial-buccal (MB) cusp of the upper first molar (U6).
Palatal Plane to Mandibular Plane Angle (PP_MP)	Angle (degrees) measured between palatal plane (ANS-PNS) and mandibular plane (Go-Me).
Lower Facial Height (LFH)	Distance (mm) measured from anterior nasal spine to menton.
Total Anterior Facial Height (TFH)	Distance (mm) measured from nasion to menton.

### STATISTICAL ANALYSIS

The evaluator randomly selected and retraced 10 sets of records (5 clear aligner and 5 traditional fixed appliance subjects) one month after the initial measurements were recorded. Intraobserver systematic errors between the replicate scans were described as mean differences and statistically compared with paired t-tests. Intraobserver random error was estimated using intraclass correlation coefficients (ICCs) and method errors (√(Σd^2^/2n).^20^ Sample size calculations were not performed prior to the study. 

The measurements were transferred to SPSS software (version 25.0, IBM SPSS Statistics, Armonk, NY, USA). Based on the skewness and kurtosis statistics, the variables were judged normally distributed. Paired *t*-tests were used to evaluate changes over time (i.e., differences between pre-treatment and post-treatment). T-tests were used to compare the groups. Box plots were used to characterize the sample using the 25^th^, 50^th^ and 75^th^ percentiles, as well median, whiskers, and outliers. Effect size was calculated using Cohen’s *d*. Linear mixed models were run to identify the effects of treatment modality (clear aligners and traditional fixed appliances) and treatment stage (pre- or post-treatment), and interaction effects of modality and stage on each measured variable. A probability level of 0.05 was used to determine statistical significance.

## RESULTS

Systematic intraobserver reliability ranged between 0.02 mm and -1.01 mm (Table 2). Five out of eight measurements (presence of posterior open bite was identical between both replicates and therefore not included) were statistically significant (Table 2). L6H showed the largest significant difference between the first and second replicates, with the first replicate 1.01 mm less than the second replicate. Mandibular plane related to cranial base (SN_MP) and to Frankfort (FMA), and palatal plane (PP_MP) also showed the first replicate smaller than the second replicate (0.17°, 0.28°, and 0.34°, respectively). The first replicate of OB was larger than the second replicate (0.16 mm). Method error ranged from 0.25 mm to 1.14 mm and from 0.35° to 1.58° (Table 2). All measurements showed highly reliable interclass correlation (ICC), ranging from 0.978 to 1.000 (Table 2). Intraobserver errors were deemed to be within an acceptable range.

**Table 2:** Intraobserver systematic errors between replicates (first minus second) for each of the measurements, along with significances (Sig) and Intraobserver random errors between replicates, estimated with method errors (ME) and interclass correlations (ICC). Bold font indicates statistically significant difference.

Variable	Difference	S.D.	Sig.	M.E.	ICC
OB (mm)	0.16	0.20	0.001	0.25	0.985
SNMP (degrees)	-0.17	0.32	0.024	0.36	0.999
FMA (degrees)	-0.28	0.37	0.002	0.46	0.999
PPMP (degrees)	-0.34	0.38	0.001	0.50	0.999
L6H (mm)	-1.01	0.53	< 0.001	1.14	0.978
U6H (mm)	-0.25	0.21	0.596	0.20	0.998
LFH (mm)	0.02	0.27	0.685	0.27	0.999
TFH (mm)	0.09	0.28	0.173	0.29	0.999

Invisalign group presented older patients than brackets group (*p*= 0.012). Prior to treatment, four out of the 44 clear aligner (Invisalign) patients and one out of the 22 traditional fixed appliances (brackets) patients exhibited posterior open bites on their lateral cephalographs. At the completion of treatment, 7 (31.82%) traditional fixed patients and 17 (38.64%) of the clear aligner patients exhibited a posterior open bite. Regarding the overbite, approximately 75% of the subjects in each group presented normal overbite prior to the treatment. Invisalign group presented two patients with anterior open bite (1.90 mm and 1.60 mm) and Brackets group had one (0.60 mm).

**Figure 3:**
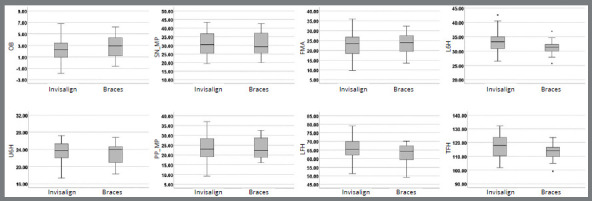
*Box plots* providing information about sample distribution, skew, and range of data for each of the eight measurements pretreatment. The upper and lower boundaries of the rectangle indicate the upper and lower quartiles, respectively. The line inside the rectangle indicates the median. The distance between the median and the quartile indicates the skew of the data. The whiskers extending from the box indicate the extreme values.

Statistical analysis showed significant difference between groups only for one out of eight variables measured (Table 3). In comparison to the Brackets group, the Invisalign group initially had larger lower molar height (≈2.0 mm). In general, Invisalign group showed slightly larger dispersion than Brackets group (Fig 3).

**Table 3:** Comparison of pretreatment values between subjects treated using clear aligners and subjects treated using conventional fixed appliances (brackets).

	Invisalign		Brackets		
Variables	Mean	SD	Mean	SD	Prob.
OB	2.33	1.84	2.71	1.90	.427
SN_MP	30.73	6.64	30.71	6.86	.990
FMA	22.65	5.72	23.42	5.70	.605
L6H	33.25	3.50	31.23	2.36	.017
U6H	23.32	2.32	23.15	2.22	.779
PP_MP	23.21	6.24	23.16	5.32	.977
LFH	65.40	6.06	63.15	5.48	.147
TFH	116.98	8.01	113.40	6.36	.073

Bold font indicates significant difference.

Regardless of pretreatment measures, both treatment modalities produced significant differences (Table 4). OB decreased in the Brackets group (1.15 mm) and remained unchanged in the Invisalign group (0.09 mm). SN_MP and FMA increased in the Invisalign group (0.43° and 0.53°, respectively) and remained the same in the Brackets group. Facial height increased in both groups. LFH increased 0.52 mm in the Invisalign group and 0.79 mm in the Brackets group. TFH increased from 116.98 mm to 117.78 mm (0.80 mm) in Invisalign patients, and from 113.40 mm to 114.14 mm (0.74 mm) in Brackets patients.

**Table 4:** Comparison of changes in subjects treated using clear aligners and subjects treated using conventional fixed appliances ( Brackets ). Negative values indicate decreasing and positive values indicate increasing over time; difference was calculated using clear aligners minus conventional fixed appliances.

	Invisalign			Brackets			Invisalign vs. braces (diff.)		
Variables	Mean	SD	Prob.	Mean	SD	Prob.	Mean	SE	Prob.
OB	-0.09	1.09	0.584	-1.15	1.61	0.003	-1.06	0.38	0.009
SN_MP	0.43	1.07	0.011	0.01	0.82	0.959	0.42	0.26	0.113
FMA	0.53	1.02	0.001	-0.00	0.79	0.979	0.53	0.25	0.038
L6H	0.17	0.61	0.068	0.63	0.76	0.001	-0.46	0.17	0.010
U6H	0.06	0.70	0.578	-0.14	0.46	0.183	0.20	0.16	0.240
PP_MP	0.02	1.13	0.905	0.17	1.03	0.454	-0.15	0.29	0.609
LFH	0.52	0.93	0.001	0.79	0.85	<0.001	-0.27	0.24	0.261
TFH	0.80	0.99	<0.001	0.74	0.93	0.001	0.06	0.25	0.817

Bold font indicates significant difference.

**Figure 4:**
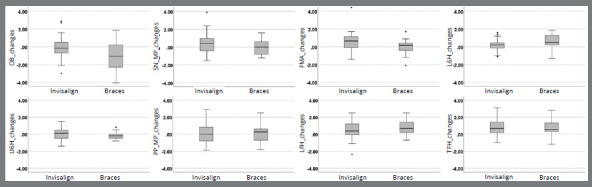
*Box plots* providing information about sample distribution, skew, and range of data for the changes due to orthodontic treatment of each of the eight measurements.

Treatment modality produced significant group change differences (Table 4). OB decreased more in the Brackets group than in the Invisalign group (1.06 mm). Relative to FMA, Invisalign patients showed greater backward rotation (0.53°). L6H increased more in the Brackets (0.63 mm) group than in the Invisalign group (0.17 mm). The Brackets group showed larger dispersion to OB changes, but in general, Invisalign group presented slightly larger amount of dispersion (Fig 4). Cohen’s effect size value of d=0.7 for OB suggested medium-to-large practical significance with a power of 0.79. Other measurements showed medium or small effect size with the differences have small to negligible practical significance (with power of 0.75 or less).

**Figure 5:**
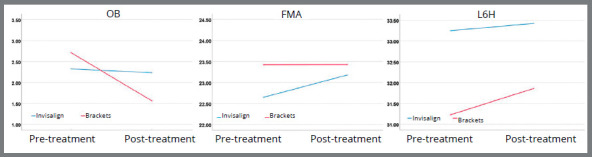
Linear mixed models identifying the effects of treatment modality (clear aligners and traditional fixed appliances) and treatment stage (pre- or post-treatment) on OB, FMA, and L6H.

OB, FMA and L6H all exhibited a statistically significant interaction between treatment stage and treatment modality (Table 5 and Figure 5). Since L6H had a significant interaction between treatment modality over the course of treatment, and because extrusion is known to be more serious in high angle patients, an additional analysis was performed to see if SN_MP or FMA values affected L6H. This analysis found that there was no significant interaction between treatment, stage and SN_MP (χ^2^= 0.536; *p*= 0.464) or between treatment, stage and FMA (χ^2^= 0.796; *p*= 0.372) on L6H.

**Table 5:** Chi-squared test (χ^2^) and probability (Prob.) of Linear Mixed Model Analysis.

Variables		Treatment (T)	Stage (S)	T:S
OB	χ^2^	0.146	7.902	9.994
	Prob.	.703	0.005	0.002
SN_MP	χ^2^	0.017	5.495	2.576
	Prob.	.897	0.019	0.109
FMA	χ^2^	0.114	9.261	4.513
	Prob.	.736	0.002	0.034
L6H	χ^2^	4.476	16.810	7.993
	Prob.	.034	<0.001	0.005
U6H	χ^2^	0.210	0.006	1.405
	Prob.	.647	0.938	0.236
PP_MP	χ^2^	<.001	0.265	0.265
	Prob.	.986	0.607	0.607
LFH	χ^2^	1.940	29.740	1.288
	Prob.	.168	<0.001	0.257
TFH	χ^2^	3.427	41.927	0.054
	Prob.	.064	<0.001	0.816
Posterior_OB	χ^2^	0.567	11.503	0.104
	Prob.	.452	<0.001	0.747

Bold font indicates significant difference.

## DISCUSSION

Vertical control of both the maxillary and mandible molars is important to control the vertical dimension in patients. Although clear aligners and traditional fixed appliances showed a good clinical vertical control of the maxillary molars, traditional fixed appliances showed slightly larger lower molar extrusion, compared to clear aligners (approximately 0.5 mm). U6H likely experienced minimal change because it is less commonly subjected to extrusive mechanics, such as elastics, than the lower molar. The lack of statistically significant change in these variables indicates that both treatment modalities are adequate at preventing excessive maxillary molar extrusion and bite opening. The slightly larger amount of extrusion of the lower molars can be observed as a side effect of elastics in the traditional fixed appliance group, and it agrees with the idea that Invisalign does not apply extrusive forces unless they are prescribed in the ClinCheck. Comparing clear aligners to fixed appliances, Garnett et al.^21^ showed also a good vertical control of the mandibular molars, with no significant difference between groups; but the authors reported the use of various auxiliaries such as lower lingual holding arches and miniscrews to treat anterior open bites. Analyzing only patients presenting open bite treated with clear aligners, Moshiri et al.^16^ showed a slightly intrusion of the mandibular molars by 0.6 mm. Comparisons are problematic because of differences in the design of the studies.

Anterior overbite decreased in traditional fixed patients and did not change due to treatment in the clear aligner patients. Buccal-lingual movement of the incisors can influence the overbite due to relative intrusion/extrusion. Patients with deeper bites or more crowding require more flaring or interproximal reduction (IPR) to align the teeth. While not a primary measure of this study, interincisor angle decreased significantly (3.44°) in the clear aligner group and did not change in the traditional fixed appliance group (6.69°). This observation could be due to treatment plan and/or patient selection; several patients treated with clear aligner therapy received interproximal reduction, allowing a better control of the incisor proclination. Studies have shown no differences related to overbite, maxillary incisor angulation, mandible incisor angulation, and interincisor angle between clear aligners and fixed appliances,^21^ or decreasing open bite in patients treated with Invisalign.^16^ In addition, Invisalign is reported to have difficulty achieving root torque,^11^ ^-^ ^13^ that should provide controlled tipping.

Although anterior facial height and lower facial height increased in both treatment modalities at the same level (differences smaller than 0.30 mm), only the clear aligner group showed slightly backward rotation of the mandible (FMA). Therefore, it is difficult to claim clinical significance. Mandibular plane is vulnerable to tracing errors due to patient positioning, landmark identification errors, and bilateral structures, making it difficult to identify in a standardized way, and this could lead to the outcome reported. When clear aligner therapy and traditional fixed appliance therapy were compared in open bite treatment, the literature showed the same changes for mandibular plane (0.60°) and anterior lower facial height (0.36 mm) for patients treated with clear aligners and traditional fixed appliances.^21^ Analyzing open bite treatment of patients under Invisalign therapy, Moshiri et al.^16^ showed small decreasing of the lower anterior facial height (1.5°) and of the mandibular plane angle (0.9°).

Presence of a visible posterior open bite significantly increased over the course of treatment in both groups. A posterior open bite was seen more frequently in post-treatment radiographs, in both the clear align group and the traditional fixed appliance group. This phenomenon is likely due to anterior interferences keeping the posterior bite out of occlusion, since intrusion of the molars was not measured in the majority of the records. It should be expected that the areas of occlusal contact increase during retention phase.^22^ ^,^ ^23^

Out of the treatment variables selected, overbite, FMA and lower molar height exhibited significant interactions between treatment stage and treatment modality. This means that the changes in pre- and post-treatment values were dependent on the treatment method (Brackets or Invisalign). L6H was analyzed further by comparing the effect of the patient’s SN_MP and FMA on its values. Analysis revealed that there was not an interaction between L6H values and the patient’s SN_MP or FMA for either treatment group. In hyperdivergent patients, there is a greater desire to control molar extrusion to prevent the mandibular backward rotation.

This study is not without limitations. Although no vertical movement of the molars were planned, the proclination of the mandibular incisors through mechanics, such as reverse curve of Spee, could affect the vertical molar position. Information about treatment mechanics (i.e. elastic use) and treatment duration was not provided for each patient. This information could be correlated to certain changes observed over the course of treatment. There is significant risk for case selection bias in this retrospective analysis. Aligner therapy has become more popular with patients and clinicians, but fixed appliances are often reserved for adults with more complicated initial malocclusions. A prospective, randomized, controlled trial would be a beneficial follow-up study to support or refute the findings reported in this study comparing clear aligner therapy and traditional fixed appliance therapy. One should keep these limitations in mind and the results should be carefully interpreted, and the generalization of the results may be limited.

## CONCLUSIONS

Within the limitations of this study, the following conclusions can be drawn:

1. Anterior overbite decreased more in the traditional fixed appliance therapy than clear aligner therapy.
2. Traditional fixed appliance therapy promoted a slightly larger lower molar extrusion than clear aligner therapy.
3. Clear aligner therapy produced slight mandibular backward rotation.
4. Both therapies (clear aligners and traditional appliances) increased the total facial height and lower facial height.
5. The clear aligner therapy did not provide a better vertical dimension control than traditional fixed appliance therapy adult patients.
